# The flow separation delay in the boundary layer by induced vortices

**DOI:** 10.1007/s12650-016-0396-0

**Published:** 2016-10-04

**Authors:** Ishtiaq A. Chaudhry, Tipu Sultan, Farrukh A. Siddiqui, M. Farhan, M. Asim

**Affiliations:** 1 School of Mechanical Aerospace and Civil Engineering, University of Manchester, Manchester, UK; 2 Department of Mechanical Engineering, Hanyang University, Seoul, Republic of Korea; 3 Department of Engineering Science, University of Oxford, Oxford, UK; 4 Department of Mechanical Engineering, University of Engineering and Technology Lahore, Lahore, Pakistan

**Keywords:** Boundary layer, Flow separation, Streamwise vortices, Synthetic jet actuator

## Abstract

**Abstract:**

A series of experiments involving the particle image velocimetry technique are carried out to analyse the quantitative effectiveness of the synthesized vortical structures towards actual flow separation control. The streamwise vortices are synthesized from the synthetic jet actuator and introduced into the attached and separating boundary layer developed on the flat plate surface. Two types of actuators with different geometrical set-ups are used to analyse the evolution of vortical structures in the near wall region and their impact towards achieving separation delay in the boundary layer. First, a single circular jet is synthesized by varying actuator operating parameters and issued into the boundary layer to evaluate the dynamics of the interaction between the vortical structures and the near wall low momentum fluid in the separated region. Second, an array of jets has been issued into the artificially separated region to assess the effectiveness of various vortical structures towards achieving the reattachment of the separated flow in the streamwise direction.

**Graphical abstract:**

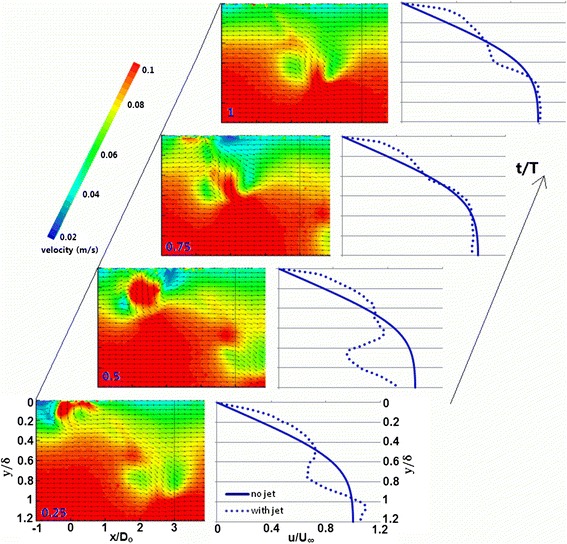

## Introduction

On an aircraft wing, the flow separation occurs when the boundary-layer travels far enough against an adverse pressure gradient. The velocity of the particles nearest to the surface falls almost to zero. The boundary-layer flow becomes detached from the surface and instead takes the forms of eddies and vortices and results in enlarged drag, particularly pressure drag. This paper explains the novelty of the synthetic jet actuator (SJA) towards achieving the reattachment of boundary-layer separation and, hence, flow separation control on aircraft wings.

By employing the SJA, the jet is synthesized from the ambient fluid by a forced periodic excitation of the diaphragm to produce a train of consecutive streamwise vortices that interact with the boundary layer to exchange momentum with the relatively less energetic near wall fluid. The vortical structures are produced over a broader range of length and timescale, and their exclusive attributes make them attractive fluidic actuators for a number of flow control applications. Smith et al. (1999) and Mallinson et al. (1999) used piezoelectric diaphragm actuators, since they are easy to build and could be operated over a broader range of frequency. In this work, the piston type actuator is used, as the main focus is on the evolution of the vortical structures and their subsequent interaction with the boundary layer.

Amitay et al. (2001) demonstrated the suppression of separation on an unconventional symmetric aerofoil. The control jet is synthesized from two side-by-side rectangular actuators parallel along spanwise dimensions. The Reynolds number was calculated based on the chord profile and was kept in the range 3.1 × 10^5^ to 7.25 × 10^5^. For inactive actuators, the aerofoil stalled for *α* > 5°, and when the actuators were active, fully attached flow was achieved for up to *α* > 17.5°. As a result of flow reattachment, or delay of separation, a substantial 100 % increase in lift was achieved and up to 45 % decrease in pressure drag was observed. For *α* > 17.5°, the lift was increased, however, the enhancement in lift was also accompanied by an increase in drag. Tensi et al. (2002) used piston type actuator with a slot opening on a cylindrical surface and observed that the separation line had been considerably pushed back downstream when the actuator was active. In the smoke visualization experiments, Gilarranz and Rediniotis (2001) used similar slot-exit actuator deployed on the upper surface of NACA0015 aerofoil with varying angle of attacks. At 20° angle of attack and with the actuators inactive, the separation occurred at the leading edge, and when the actuators were active, the separation line was pushed back downstream up to 70 % of chord length.

Certainly the effectiveness of synthetic jet actuators towards flow separation control has long been recognized in laboratory experiments. However, the fluid dynamics involved in the interaction of vortical structures with the boundary layer ultimately causes the reattachment that is yet far from fully understood. The flow visualization by Ishtiaq and Zhong (2014) and the liquid crystal and PIV investigations by Jabbal and Zhong (2010) revealed different types of vortical structures formed under varying operating parameters when the actuator is deployed in the cross-flow zero-pressure gradient boundary layer. Jabbal and Zhong (2010) were able to come up with some quantitative analysis based on liquid crystal and PIV measurements and concluded that stretch vortex rings (SVR) were the most desirable structures to delay separation line. Zhong et al. (2005) proposed, however, without any quantitative analysis, that hairpin vortices (HP) were the most effective structures, since they produced two parallel streaks of enhanced shear stress as a result of counter rotating legs. On the other hand, the most recent PIV measurements (Ishtiaq and Zhong 2013) carried out on the spanwise plane revealed that the lateral wall shear enhancement was the maximum when the tilted vortical rings (TVR) were issued into the boundary layer.

Therefore, it is essential to carry out further investigation into the fluid dynamics involved in the interaction of varying vortical structures with the near wall fluid. For more reliable and authentic results, the effectiveness of the various types of vortices needs to be evaluated in the actual artificially produced separation region. Furthermore, it has been observed that the SJA operating parameters seem to vary to produce similar vortical structures under similar free stream conditions.

## Experimental set-up

**Fig. 1:**
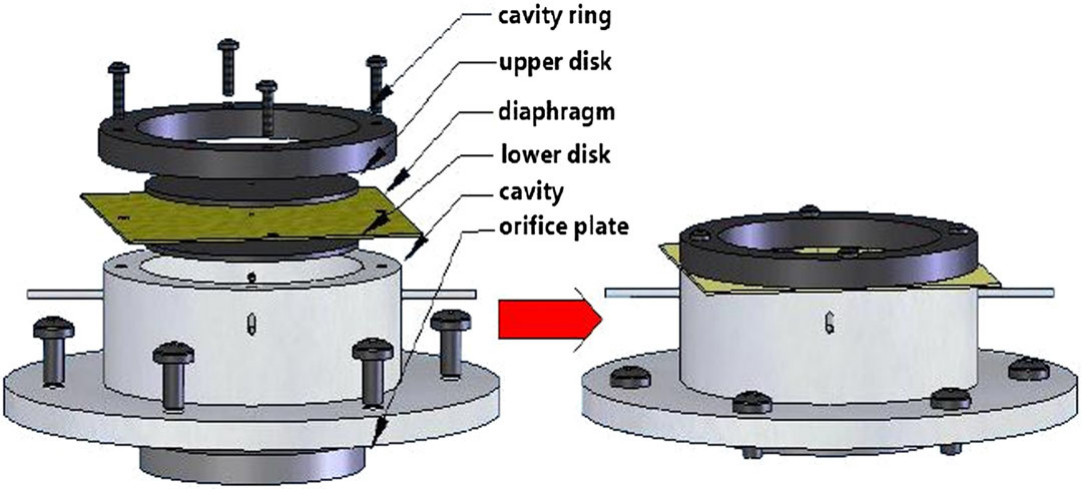
Circular synthetic jet actuator assembly

A bench-top SJA rig was built on a tilted water flume at the Hydraulics Laboratory Pariser, the University of Manchester. The actuator consists of a diaphragm located at the top of the cavity and a circular orifice on the bottom face opposite to the diaphragm. The actuator design (Fig. 1) is such that to and fro movement of the diaphragm can be measured conveniently and the specific size of the cavity is kept fixed with the following dimensions: internal diameter, *D*_c_ = 45 mm; height, *H* = 25 mm.

**Fig. 2:**
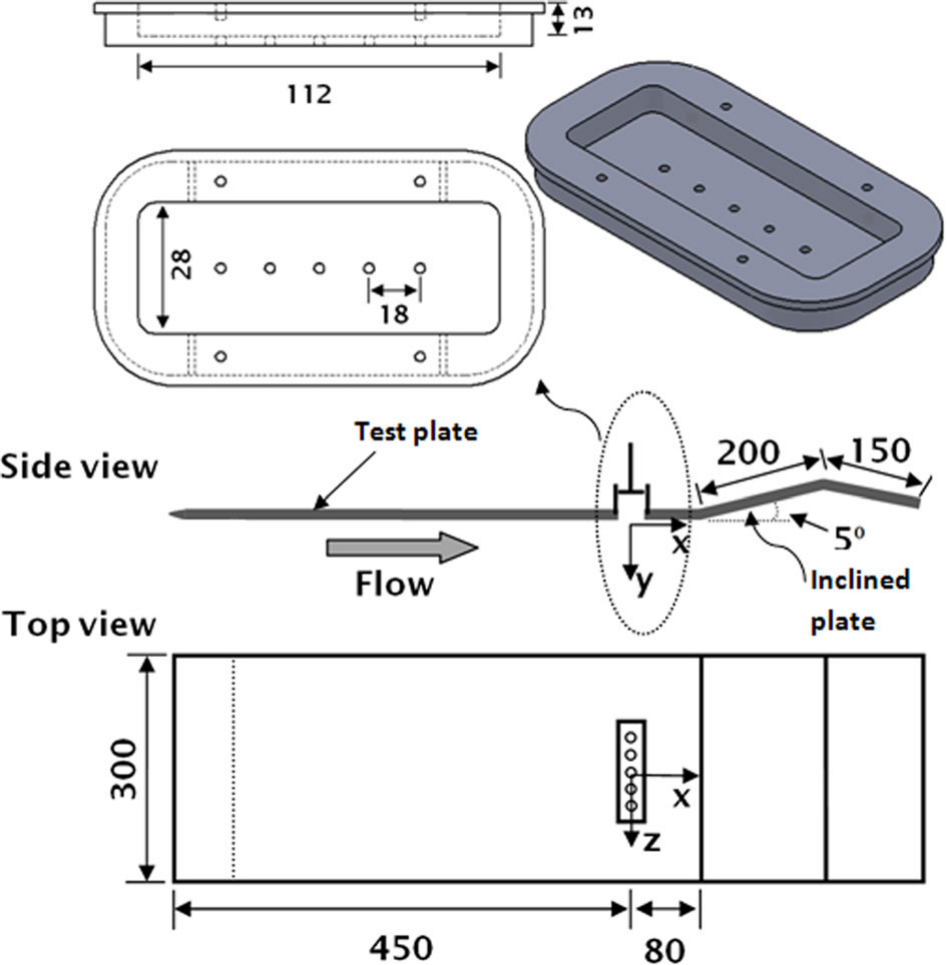
Test plate for separated flow and rectangular cavity (mm)

For the second series of experiments, the artificial separation is generated on an inclined plate attached to the trailing edge of the test plate. The rectangular cavity is used to accommodate an array of orifices (Fig. 2). The test plate is made from a 5 mm-thick sheet of aluminium (Al HE30) with the super elliptical leading edge with 1:5 nose thicknesses to length ratio, to avoid any premature transition at the leading edge. The free stream velocity is kept constant at 0.1 m/s and is measured first by a dye visualization technique and second confirmed using a Nixon Streamflow vane anemometer. The main components of the PIV system are Nd: YAG pulse lasers (with a maximum power of 135 mJ, repetition rate up to 15 Hz, and the laser pulse width 3–5 ns), TSI synchronizer, and a Hitachi KP-F120 partial scan CCD camera with a maximum available resolution of 2048 × 2048 pixels, 12-bit intensity dynamic range, and a frame rate of up to 16 per second.

Along the streamwise plane (*x*–*y*) normal to the plate surface, a laser light sheet is produced through the glass-bottomed floor of the test section, whilst positioning the camera, such that its optical axis is facing the side wall of the flume and the laser sheet. The suitable laser light sheet with desired thickness and width is formed using a combination of cylindrical and spherical lens and was directed to the required area on the test plate using a 50 mm-thick laser mirror mounted at 45° on a support. For safety purpose, the ends of the laser head and sides of the glass flume were enclosed and fully covered using black paper to avoid any unwanted laser leakage.

From the basis of the dynamic incompressible flow model established by Tang and Zhong (2006), the time averaged blowing velocity over the cycle is given by

1 $$\overline{U}_{\text{o}} = \, \frac{1}{T}\int_{0}^{T/2} {\widetilde{u}}_{\text{o}} \left( t \right){\text{d}}t = f\Delta \left( {\frac{{D_{\text{c}} }}{{D_{\text{o}} }}} \right)^{2} .$$

According to the slug model (Glezer 1988), the stroke length (length of the fluid slug) is now defined as

2 $$L_{\text{o}} = \int_{0}^{T/2} {\widetilde{u}}_{\text{o}} \left( t \right){\text{d}}t,$$

3 $$L = \frac{{L_{\text{o}} }}{{D_{\text{o}} }} = \frac{{\overline{U}_{\text{o}} T}}{{D_{\text{o}} }} = \frac{{\overline{U}_{\text{o}} }}{{fD_{\text{o}} }},$$

where *‘f’* (Hz) is the excitation frequency and can be non-dimensionalised in Strouhal number as

4 $$St = \frac{{fD_{\text{o}} }}{{\overline{U}_{\text{o}} }} = \frac{1}{L}.$$

The Reynolds number is related with the stroke length and can be expressed as follows:

5 $$Re_{L} = \frac{{\overline{U}_{\text{o}} L_{\text{o}} }}{\nu } = Re\frac{{L_{\text{o}} }}{{D_{\text{o}} }} = Re_{L} .$$

For incompressible isothermal flow, the boundary-layer thickness is non-dimensionalised by the orifice diameter

6 $$d = \frac{\delta }{{D_{\text{o}} }}.$$

In addition to the key parameters that is ‘*L’* and ‘*Re*_*L*_’, the jet velocity-to-freestream velocity ratio (VR*)* is considered and is given by

7 $${\text{VR}} = \frac{{\overline{U}_{\text{o}} }}{{U_{\infty } }}.$$

The smallest frequency used in the experiments is 1 Hz which is at least two times greater than Tollmien–Schlichting wave, so the effect of SJA does not influence the stability of the boundary layer. However, the stability is confirmed by calculating the indifference Reynolds number to be ≈383 which is not exceeding the minimum value of 520.

## Results and discussion

### Test conditions

**Fig. 3:**
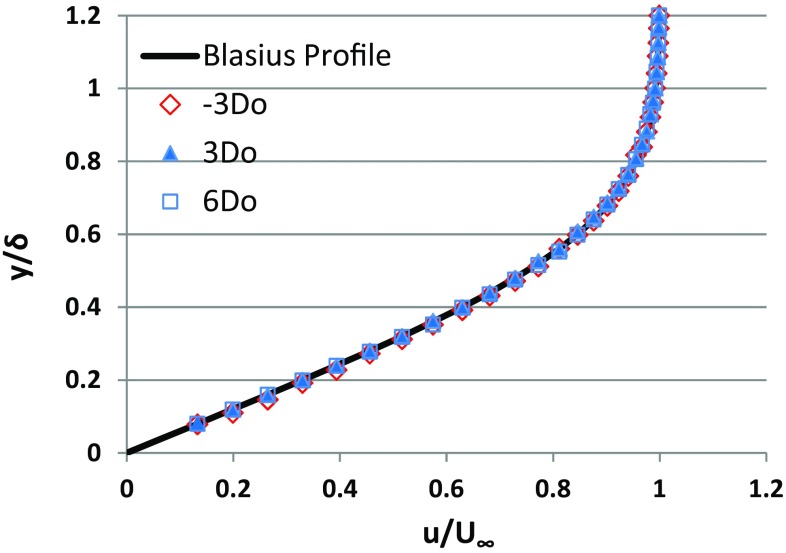
Boundary-layer profile

The velocity profiles are drawn at three different locations deemed critical for final measurements. With the no slip condition at the wall, a good match with Blasius profile is met to confirm the zero-pressure gradient nature of the boundary layer (Fig. 3). The profiles are extracted from the velocity field obtained by averaging 500 frames taken at 6 Hz with 0.36 × 0.36 mm^2^ interrogation area.

### Vortex impact on the boundary layer

**Fig. 4:**
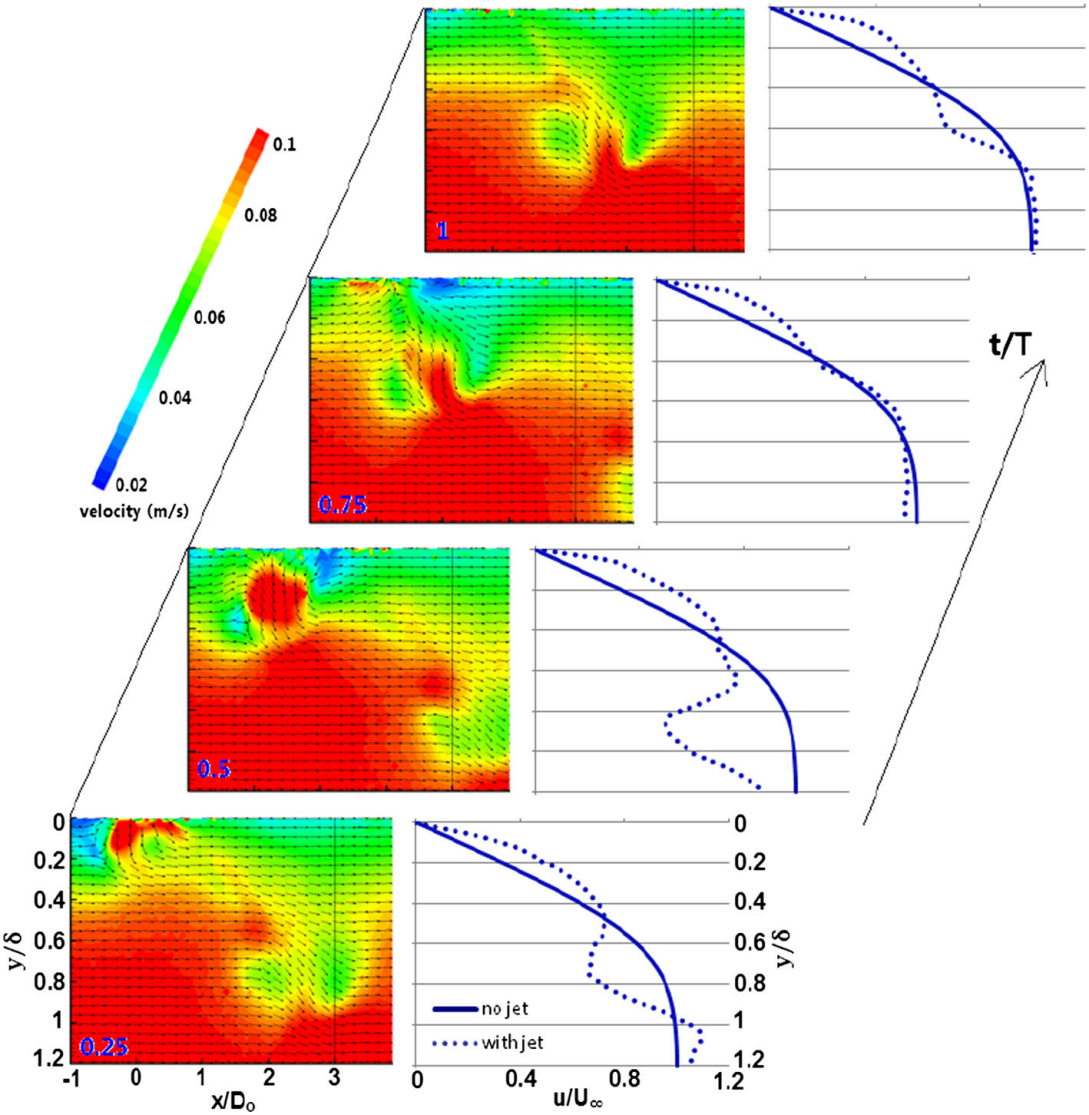
Transient variation of centerline phase-averaged velocity profile for passing TVR

The eventual impact of the passing vortex in the boundary layer can be apprehended by the way of quantitatively evaluating the transient effect on the undisturbed boundary-layer profile. Hence, the transient variation of the centerline phase-averaged velocity profiles for tilted vortices has been undertaken. The velocity contours are shown in Fig. 4 with an overlay of uniformly distributed velocity vectors to serve supplementary interpretation.

For four phase points evenly distributed over the actuation cycle, the profile is shown in Fig. 4 for tilted vortex ring. Over the complete course of actuation cycle, the velocity increment near the wall region remains fairly enhanced suggesting a non-intermittent influence. At *t*/*T* = 0.25, where ‘*t*’ is the time from start of actuation cycle, the deficit is quite significant caused by the downstream branch of the vortex and the deficit region is far away from the wall, as the vortex has had travelled far away from the wall surface by virtue of larger stroke length. The abrupt decrement from *y*/*δ* = 0.5 to 0.78 is due to the upper side of the downstream branch having opposite sense of rotation relative to the local velocity. Similarly, the lower part of the downstream branch exhibits the same sense as the local velocity hence suggesting a far steeper velocity gradient from *y*/*δ* = 0.78 to 1.05. Such a gradient subsequently results in the velocity over shoot in the region outside the boundary layer.

At *t*/*T* = 0.50, the upstream branch approaches the measurement area and it displays clockwise vorticity having similar sense in the upper part as the local velocity and the opposite sense in the lower part. From *y*/*δ* = 0.4 to 0.65, the abrupt velocity increment is due to the upper part of the upstream branch. However, the lower part of the upstream branch decelerates the local velocity from *y*/*δ* = 0.65 to 0.85. Further deep towards the outermost part of the boundary layer, the velocity increment is due to the upwash of the fluid. At *t*/*T* = 1, the vortex of the previous cycle has passed the measurement plane, as the newer structure approaches. There appears a velocity increment near the wall region and then a decent decrement at about *y*/*δ* = 0.2 caused by the passing trailing secondary and tertiary vortices. Second, the velocity gradient near the wall region is larger in the case of tilted vortices than the stretched and hairpins, where the velocity gradient is generated by the upwash of the fluid resulted from the counter rotating legs. On the other hand in TVR’s, when the primary vortex has had moved away from the wall, the near wall velocity gradient could still be seen which is far significant and is generated by the trailing counter rotating tertiary vortices inducing a low momentum fluid downwash towards the wall. The tertiary vortices are, in fact, the trailing streamwise vortices with the opposite sense of rotation to secondary vortices thus result in downwash of the fluid. The appearance of secondary and tertiary vortices is more evident in the dye visualization technique (Ishtiaq and Zhong 2014).

### Off-centre velocity distribution

**Fig. 5:**
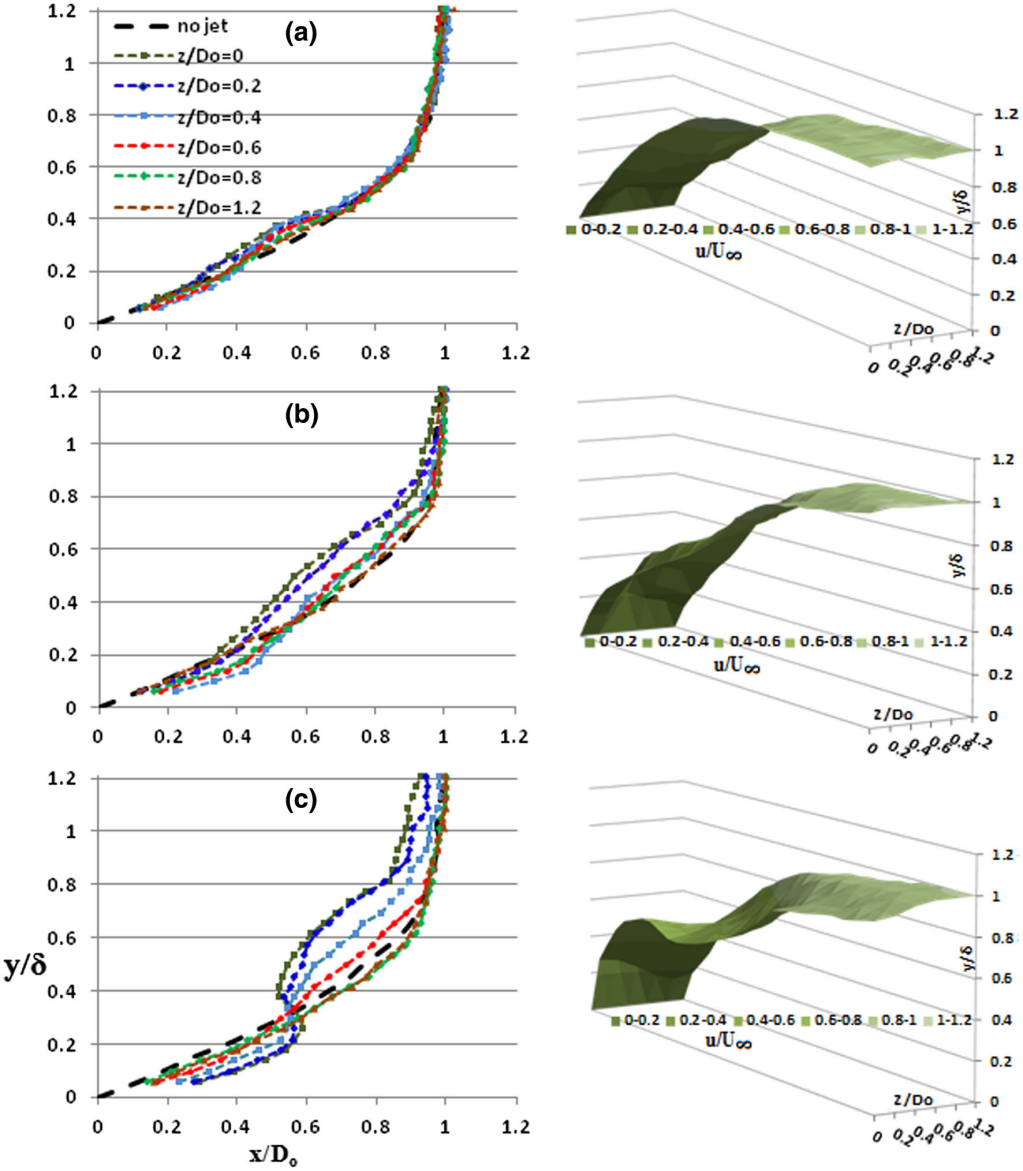
Off-centre line time averaged velocity profiles for a passing vortex for **a** HP, **b** SVR, **c** TVR

The lateral influence of the vortical structures is analyzed by taking velocity profiles at off-centre locations, as shown in Fig. 5. The velocity profiles are also shown in a 3-D chart to aid the visualization effects of the profile modification. For HP, the influence is fairly visible in the near the wall region up to a significant spanwise width. The maximum velocity gradient is attained at about *z*/*D*_o_ = 0.4, where the outboard of the hairpin leg coincides having the maximum fluid downwash towards the wall. From 3D chart, it appears that the maximum disturbed plane is fairly away from the central plane. Further outboard the deficit area tends to reduce, as the starting point on the profile is pushed farther away from the wall to a larger value of ‘*y*/*δ*’. Hence, there is a considerable reduction in the deficit area, as the near wall region is being filled up. Such a redistribution of velocity deficit is, in fact, a measure of the redistribution of momentum within the boundary layer.

For stretched vortex rings (SVR), the largest velocity deficit appears closer to the central plane in the outer boundary-layer region which is the direct consequence of the low momentum fluid lift up due to the interaction of the vortex head and the counter rotating legs with the local fluid. A stretched vortex clearly produces a stronger inflexion and larger deficit than the hairpins. Away from the centerline, such as *z*/*D*_o_ = 0.4 and 0.6, there appears an obvious reduction in the velocity deficits and the deficit shifts farther away from the wall filling up the gap towards the undisturbed profile. Simultaneously, the gradient is increasing sharply in the near wall region, as the velocity increases abruptly from zero at the wall. Similar to hairpins, the maximum gradient occurs at *z*/*D*_o_ = 0.4 and afterwards the gradient tend to decreases towards the farthest plane.

The tilted vortices influence the boundary layer up to the very edge and beyond, as the deficit area widens up quite significantly. In the near wall region, the gradient seems rather enhanced especially on the closer locations to the central plane. The maximum gradient occurs at the central plane that corresponds to the fluid downwash induced by the tertiary vortices that remain closer to the wall. The gradient is steepest and is shifted to the central plane, where the maximum momentum redistribution and fluid mixing occur eventually. The change in velocity profiles is significant around the central plane. First, for central plane, there is an abrupt variation at *y*/*δ* = 0.4 caused by the secondary trailing vortex and second at *y*/*δ* = 0.9 due to the primary vortex. There appears a gradual reduction in the maximum gradient achieved corresponding to the movement away from the region of maximum downwash induced by the trailing tertiary vortex pair. Similarly, there is a spanwise velocity increment that coincides with the gradual movement from the inboard side of the trailing secondary vortex pair responsible to induce a flow away from the wall. Moreover, towards the boundary-layer edge, a localized region of velocity deceleration is noticed that is produced by the interaction of the primary tilted vortex that tends to protrude out of the boundary layer.

### Actual control effect

For the second set of experiments, the design of flat plate is altered by attaching an inclined plate to the trailing edge. It is inclined upward at an angle of 5° to generate the artificial separation on the inclined surface, as shown in Fig. 2. The joint between the two plates is filled up with a thin rubber sheet to provide a smooth flat surface to avoid any premature separation at the joint. An array of jets have been generated from five orifices from a multiple hole orifice plate and issued into the separated region to evaluate the effectiveness of the vortical structures towards flow separation delay on the surface. At the end, a third plate 150 mm long is attached and deflected downwards appropriately to ensure stagnation point at the leading edge. The measurement area is illuminated with a laser sheet that forms the measurement plane. The laser sheet is kept parallel to the inclined plate and is kept 1 mm away from the surface to reduce any unwanted deflections. Although the SJA geometrical dimensions have been changed in terms of cavity volume and orifice diameters, the non-dimensional parameters like VR, *Re*_*L*_, and *L* are kept similar to those mentioned earlier.

**Fig. 6:**
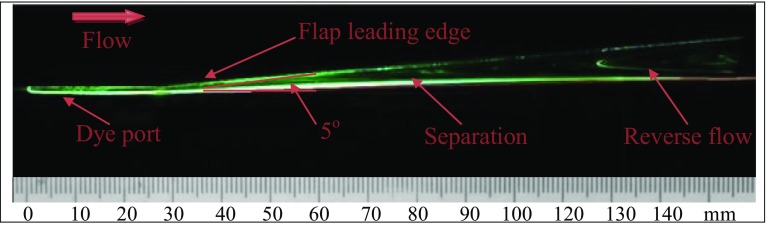
Reverse flow at the inclined plate

**Fig. 7:**
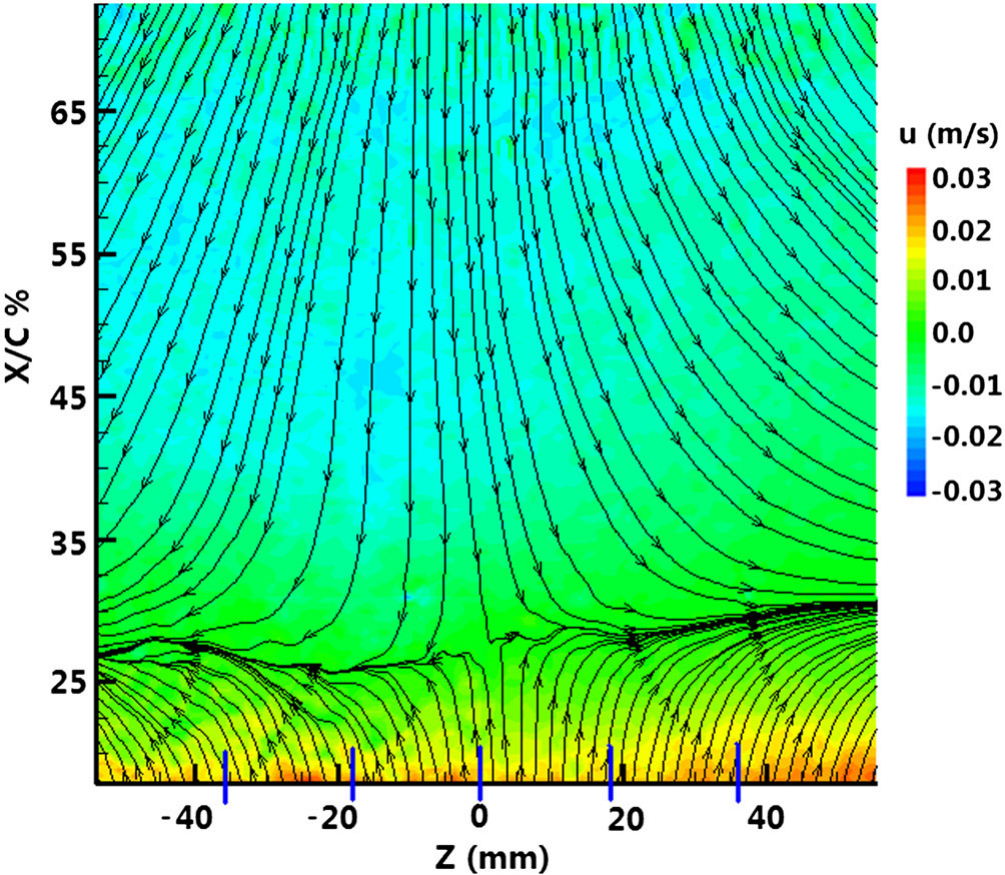
Baseline streamwise velocity contours

**Fig. 8:**
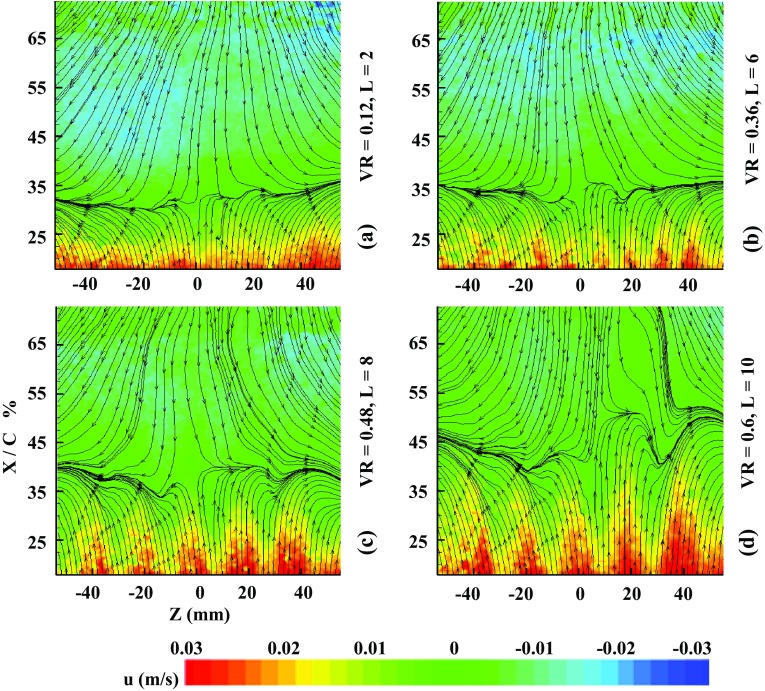
Streamwise streamlines and velocity contours for controlled case at *f* = 2

In Fig. 6, the reverse flow or flow detachment can be seen on the inclined plane. Figure 7 shows the contours of time-average velocity in the lateral plane along with the layout of streamlines where the flow is from bottom to top. The blue lines mark the orifice spanwise locations. For the inactive actuator, the flow separates at about 27 % *X*/*C* (‘*C*’ is the streamwise inclined plate length), where the upcoming streamlines seem to deflect sideways. After the separation (known as the baseline), the streamlines are pointing downwards indicating the reverse flow. For controlled cases, the footprints induced by the synthetic jet are shown in Fig. 8 when the actuator is operated at *f* = 2. The separation line has been pushed back considerably even for the lowest ‘VR’ (case a). Note that at this ‘VR’ value, the hairpins just start to form as identified in parameter map (Ishtiaq and Zhong 2014). Before the formation of hairpins, there is no noticeable delay in the separation line. Hence, for the initial control effect, the SJA must be operated in the region, where there is actual formation of the hairpins on the parameter map.

The separation line is pushed back further (case b), as the hairpins are becoming stronger and more capable to withstand the near wall shear. Therefore, they interact strongly with the local low momentum fluid in the near wall region. The high velocity streaks are becoming more prominent, as the separation line is pushed further back. Finally, the vortices are turned to tilted vortex rings, as the ‘VR’ is increased (case c, d). Because of their tendency of protruding out of the boundary layer, the primary vortices do not affect the separation delay significantly. For tilted vortices case, the separation delay is caused by the trailing induced vortices that remain essentially within the boundary layer.

**Fig. 9:**
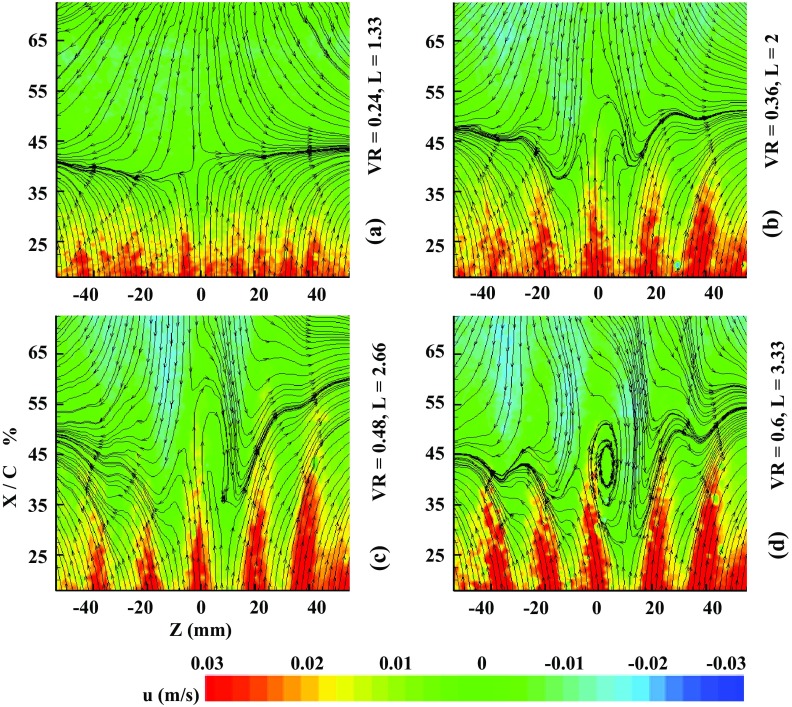
Streamwise streamlines and velocity contours for controlled case at *f* = 4

**Fig. 10:**
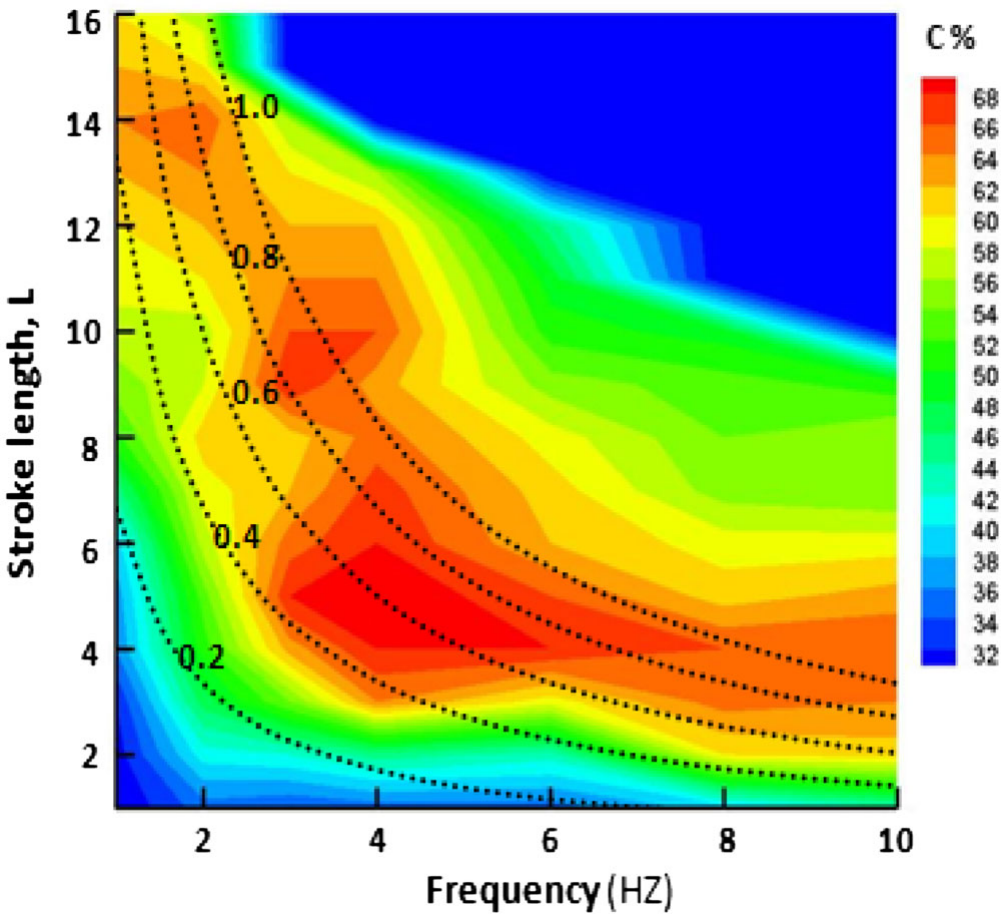
Contours of overall separation control effect for active actuator

For *St* = 1.8, the footprints induced are shown in Fig. 9 when the actuator is operated at *f* = 4. At smaller VR (case a), the hairpins produce a pair of high-speed streaks similar to the stretched vortices. At slightly larger VR, there is only one single high-speed streak (case b) and the separation line is pushed back to about 45 % of *C*. As the velocity ratio increases, the flow pattern seems to deviate in lateral direction. For tilted vortices (case c, d), the high-speed streaks are strengthening and are transformed to a single streak stretching out from each orifice. At high VR (case f), there appears a decrease in separation effect, as the tilted vortices are pushed out of the boundary layer and the downstream course of the trailing vortices is far too steep. It appears that further increase in ‘VR’ and ‘*L*’ does not help to achieve any additional streamwise delay rather the flow is becoming more swirling. Hence, it could be deduced that relatively higher ‘VR’ and ‘*L*’ would not be considered as the desirable parameters to operate the SJA for maximum flow separation control. The quantitative flow separation delay in the form of contours is given in Fig. 10. The velocity ratio plot calculated against frequency ‘*f*’ and dimensionless stroke length ‘*L*’ yields diagonal curves. The maximum delay is achieved at frequency 4 Hz and the stroke length is from 4 to 6. The novelty of SJA concerns with the fact that it consumes minimum input energy to yield the best control effect. Therefore, it must be operated at lowest possible stroke length to ensure minimum possible input energy.

## Conclusions

A series of particle image velocimetry (PIV) experiments have been conducted to evaluate the dynamics of vortical structures to confine the synthetic jet actuator operating parameters, where the best separation delay effect is achieved. The stretched vortices appear to influence the boundary layer in the similar manner as the hairpins in that the occurrence of the maximum velocity gradient in the near wall region is consistent with the fluid downwash towards the wall produced by the interaction of the counter rotating vortex legs. The stretched vortices are more likely to produce a rather fuller profile with a larger velocity deficit area across the boundary-layer thickness. Subsequently, this would appear to be capable of higher momentum redistribution of fluid from the outer part of the boundary layer towards the wall.

However, considering the quantitative evaluation, it appears that the tilted vortex rings seem to push the base line backwards to the maximum extent. It appears that secondary and tertiary vortices produced in the wake of the primary tilted vortex are the most effective structures towards flow separation delay. The tertiary vortices interact with the near wall fluid in the same way, as the primary hairpins in that they tend to force the fluid downwash towards the wall on the centre line. The effect seems more acute compared with the hairpins, as they interact with the fluid readily energized by the interaction of secondary vortices. Second, the fluid downwash on the single centre line is far more enhanced than the hairpins and stretch vortex rings (SVR) which tend to produce two streaks outboard of the vortex legs. In the artificially produced separation region, the single high-speed streaks seem to delay the separation to a maximum when compared with two streaks. The tilted vortices produced at low-velocity ratio are more effective than those produced at larger velocity ratio. At higher velocity ratio, the swirling flow around the separation line is achieved that inhibits the further delay of the baseline.

## List of symbols

*D*: Diameter of cavity or orifice, mm
*L*: Dimensionless stroke length
*U*: Characteristic velocity, m*/*s
*y*: Normal distance from wall, mm
*α*: Angle of attack
*ν*: Molecular kinematic viscosity, m^2^ */*s
*τ*: Surface shear stress, N/m^2^
*C*: The streamwise inclined plate length
*T*: Diaphragm time period
*Re*_*L*_: Reynolds number based on dimensionless stroke length
*F*: Diaphragm oscillation frequency, Hz
*St.*: Strouhal number
*x*: Streamwise distance, mm
*z*: Spanwise distance from the middle orifice
*δ*: Boundary-layer thickness, mm
*ρ*: Fluid density, kg/m^3^
*U*_o_: Space averaged jet velocity, m/s
*X*: The streamwise distance on inclined plate
∆: Peak-to-peak diaphragm displacement, mm

## Subscripts

*C*: Cavity value
∞: Freestream value
*o*: Orifice value

## Superscripts

–: Time average

## Abbreviations

HP: Hairpin vortices
SJA: Synthetic jet actuator
VR: Velocity ratio
TVR: Tilted vortex rings
SVR: Stretched vortex rings
PIV: Particle image velocimetry
